# Hyperammonemia After Lung Transplantation: Systematic Review and a Mini Case Series

**DOI:** 10.3389/ti.2022.10433

**Published:** 2022-05-03

**Authors:** Amir Y. Kamel, Amir M. Emtiazjoo, Lauren Adkins, Abbas Shahmohammadi, Hassan Alnuaimat, Andres Pelaez, Tiago Machuca, Mauricio Pipkin, Hyun-wook Lee, I. David Weiner, Satish Chandrashekaran

**Affiliations:** ^1^ Department of Pharmacy, UF Health Shands Hospital, College of Pharmacy, University of Florida, Gainesville, FL, United States; ^2^ Division of Pulmonary, Critical Care and Sleep Medicine, UF Lung Transplant Program, College of Medicine, University of Florida Health Hospital, Gainesville, FL, United States; ^3^ College of Pharmacy Liaison Librarian, Health Science Center Libraries, Gainesville, FL, United States; ^4^ Division of Cardiothoracic Surgery, UF Lung Transplant Program, University of Florida Health Hospital, College of Medicine, University of Florida, Gainesville, FL, United States; ^5^ Division of Nephrology, Hypertension and Renal Transplantation, College of Medicine, University of Florida, Gainesville, FL, United States; ^6^ North Florida/South Georgia Veterans Health System, Gainesville, FL, United States

**Keywords:** hyperammonemia, lung transplantation, ammonia, glutamine synthetase, urea cycle, ammonia scavengers, mollicutes

## Abstract

**Background:** Hyperammonemia after lung transplantation (HALT) is a rare but serious complication with high mortality. This systematic review delineates possible etiologies of HALT and highlights successful strategies used to manage this fatal complication.

**Methods:** Seven biomedical databases and grey literature sources were searched using keywords relevant to hyperammonemia and lung transplantation for publications between 1995 and 2020. Additionally, we retrospectively analyzed HALT cases managed at our institution between January 2016 and August 2018.

**Results:** The systematic review resulted in 18 studies with 40 individual cases. The mean peak ammonia level was 769 μmol/L at a mean of 14.1 days post-transplant. The mortality due to HALT was 57.5%. In our cohort of 120 lung transplants performed, four cases of HALT were identified. The mean peak ammonia level was 180.5 μmol/L at a mean of 11 days after transplantation. HALT in all four patients was successfully treated using a multimodal approach with an overall mortality of 25%.

**Conclusion:** The incidence of HALT (3.3%) in our institution is comparable to prior reports. Nonetheless, ammonia levels in our cohort were not as high as previously reported and peaked earlier. We attributed these significant differences to early recognition and prompt institution of multimodal treatment approach.

## Graphical Abstract

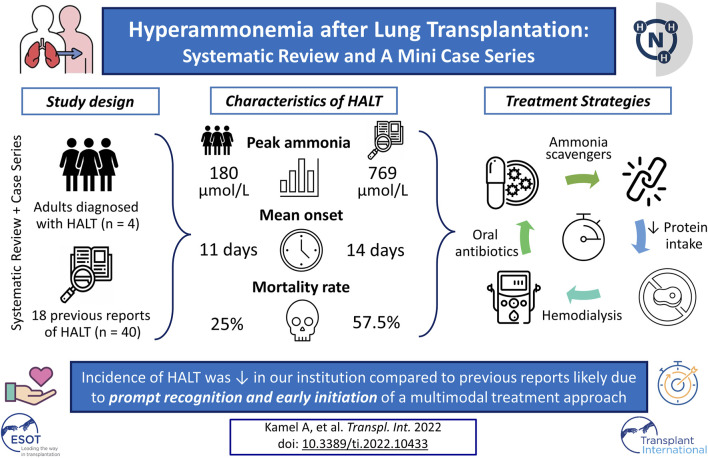

## Introduction

Hyperammonemia after lung transplantation (HALT) is a rare but often fatal complication. It manifests as an elevated serum ammonia level that leads to encephalopathy, cerebral edema, seizure, coma, cerebral herniation, and death. Reported incidence of HALT ranges from 0.99 to 4%, with fatality rates exceeding 75% (1–3).

Hyperammonemia (HA) after organ transplantation has also been described in patients who underwent bone marrow, liver, kidney, and heart transplantation (4–12). The exact etiology of HALT is unknown. Ultimately, the cause of HA may be related to excess production, decreased clearance of ammonia, or both. Disorders of glutamine synthetase (GS) have been described in patients with HALT (1,13,14). More recently, infection with urea-splitting microorganisms has been reported in patients with HA after organ transplantation (11,12,15–17).

**FIGURE 1:**
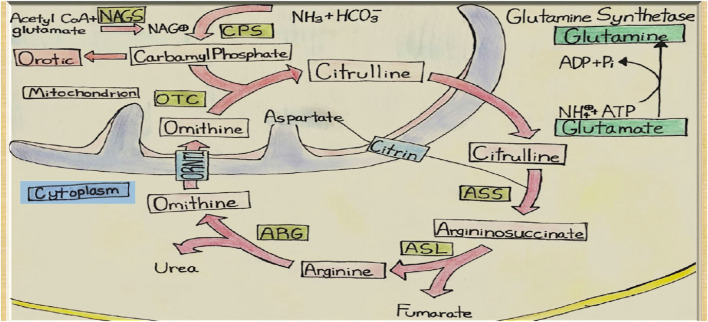
Urea Cycle and Hepatic Glutamine Synthetase. NAGS, N-acetyl glutamate synthetase; CPS, Carbamoyl Phosphate Synthetase; OTC, Ornithine Transcarbamoylase; ASS, ArgininosuccinateSynthetase; ASL, ArgininosuccinateLyase; ARG, Arginase; ORNT1, Ornithine Translocase.

This systematic review aims to explore potential etiologies and investigate if patients’ metabolic profile supports the Urea cycle (UC) pathway involvement (Figure 1). Additionally, we report our center’s successful experience managing four HALT cases, emphasizing an alternative approach to therapy. In one of the cases, we examined the liver tissue obtained at biopsy, which showed a significant downregulation of GS, suggesting a potential role for the GS pathway in HALT.

## Materials and Methods

### Data Sources

A health science librarian performed a systematic search of the medical literature on the occurrence of HA in lung transplants. An initial examination of six bibliographic databases and grey literature sources, CINAHL, Clinical Trials.gov, Cochrane Library, Embase, International Pharmaceutical Abstracts (IPA), PubMed, and Web of Science, was performed. The search strategy combined database-specific controlled vocabulary, truncated, and phrase-searched HA and lung transplantation keywords limited to English language full-text and publication dates of 1995–2020 (Supplementary Material S1).

### Study Selection Criteria

To be included in the systematic review, a study had to 1) be performed in adults that developed HALT requiring treatment, combined cases of other organs were not included, and 2) have full text available in English.

### Data Extraction

Extracted data included author name, year of publication, number of patients, time to peak ammonia level, peak ammonia level, treatment with bowel decontamination, nitrogen scavengers and renal replacement therapy, outcome, and hypothesized etiology of HA.

### Our Experience

We performed a retrospective review of all lung transplants performed at the University of Florida Health hospital between 1 January 2016, and 31 August 2018. We screened for HA episodes by extracting serum ammonia levels from the electronic medical record obtained on and after the transplantation. Hyperammonemia was defined as any plasma ammonia level higher than the upper limit of normal as previously described (18). At our center 60 μmol/L is the upper limit of normal.

In addition to hyperammonemia, our definition of HALT required meeting at least three out of the following criteria: 1) absence of cirrhosis, liver failure, or history of liver transplantation; 2) the presence of encephalopathy; 3) administration of specific treatment for HA; and 4) agreement of at least two independent reviewers (AK, AE, SC). We extracted the following data: demographic information, induction and maintenance immunosuppression used, ammonia levels, metabolic profile, baseline laboratory, treatment with bowel decontamination, nitrogen scavengers, RRT modality, and patient outcomes. The University of Florida Institutional Review Board approved this study.

### Statistical Analysis

Statistical software JMP (SAS v 15) was used to analyze the data. Descriptive analyses were applied for demographic variables and medical condition variables. Means and standard deviations were calculated for continuous variables; frequencies and percentages were calculated for categorical variables.

### Glutamine Synthetase Immunohistochemistry

**FIGURE 2:**
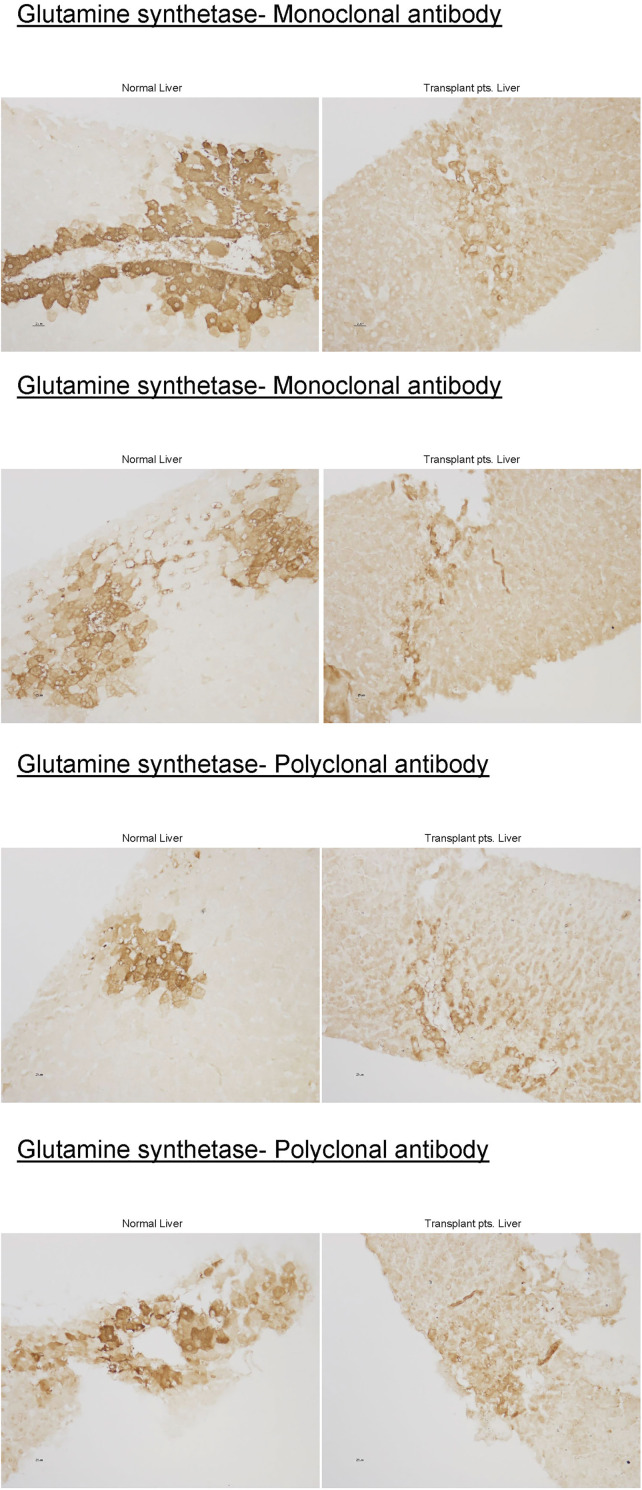
(Continued).

Liver biopsy obtained from the first case was stained for GS enzyme activity using mono and polyclonal antibodies and compared to healthy liver tissue control (Figure 2).

## Results

### Characteristics of the Systematic Review

**FIGURE 3:**
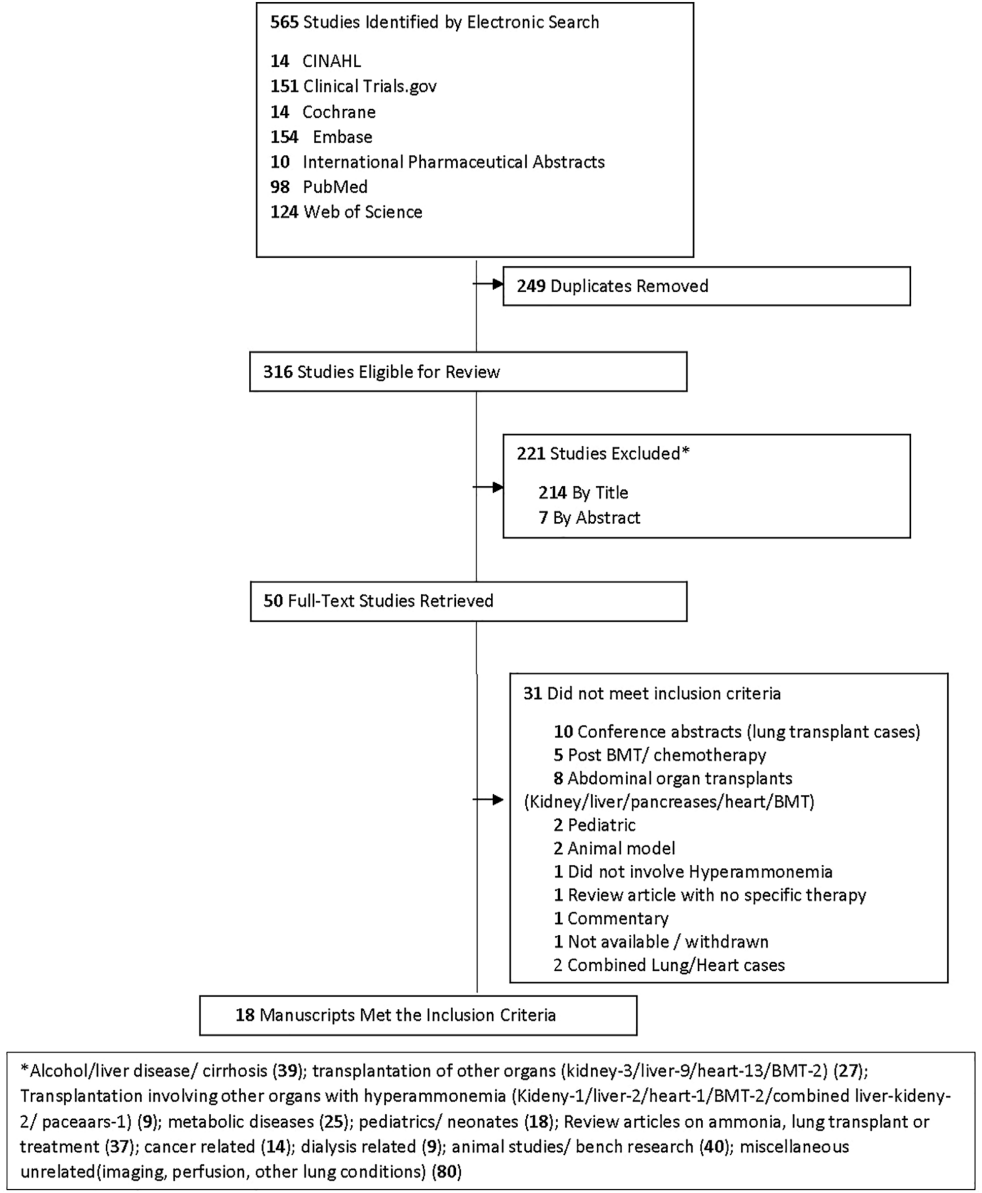
PRISMA Flow Diagram.

A flow chart of screening and selection for inclusion is presented in Figure 3. After filtering out duplicate studies, our search resulted in 18 studies, including 40 individual cases that met inclusion for full-text review. Details of previously published HALT cases are reported in Table 1. Time from transplantation to peak ammonia level ranged from 1 to 45 days (mean 14.1 days) of the 35 cases with reported values. Peak ammonia levels ranged from 55 to 5,000 μmol/L (mean 760.2 μmol/L) of the 35 cases with reported values. Of the 40 cases, 17 (42.5%) survived and 23 (57.5%) died.

**TABLE 1:** Previous Reported Cases of Post lung Transplant Hyperammonemia.

References	Gender	Case (s)	POD to peak	Peak NH_3_	Bowel decontamination	N_2_ scavengers	RRT	Outcome	Hypothesized etiology
(31)	F	1	7	3,207	L	NR	CAVHD	Died	Glutamine synthetase deficiency
(19)	F	1	35	535	L, N	SB	IHD	Died	Glutamine synthetase deficiency
(32)	M	1	1	269	L, N	AR,SB, SP	Hemoperfusion with charcoal + IHD	Survived	NR
(1)		4							
Patient 1	NR	—	29	5000	L +/− N	NR	None	Died	Idiopathic
Patient 2	NR	—	27	900	L +/− N	SB, SP	None	Died	Idiopathic
Patient 3	NR	—	5	3136	None	NR	None	Died	Idiopathic
Patient 4	NR	—	20	1800	L +/− N	SB, SP	IHD	Survived	Idiopathic
(33)	M	1	22	338	L, ME, R	AR, LC, SB, SP	CVVHD	Survived	Idiopathic
(14)	F	1	5	>1,200	L, R	NR	CVVHD	Died	*Mycoplasma hominis*
(34)	F	1	7	704	L, R	NR	CVVHD	Died	*Mycoplasma hominis*
(35)		4							
Patient 1	NR	—	NR	NR	L	SB, SP	CVVHD	Died	Idiopathic
Patient 2	NR	—	NR	NR	L	SB, SP	CVVHD	Died	Idiopathic
Patient 3	NR	—	NR	NR	L	SB, SP	CVVHD	Died	Idiopathic
Patient 4	NR	—	NR	NR	L	SB, SP	CVVHD	Survived	Idiopathic
(27)		3							
Patient 1	M	—	9	269	L, N	AA, AR, SB, SP	CVVHD+IHD	Survived	Idiopathic
Patient 2	M	—	12	330	R	AR, LC	CVVHD	Died	Idiopathic
Patient 3	M	—	9	475	L, R, ME	AR, LC, SP	CVVHD, IHD	Survived	Idiopathic
(11)		5							
Patient 1	M	—	10	291	L, R	LC, SB, SP	CVVHD	Died	*Ureaplasma urealyticum* infection
Patient 2	M	—	10	NR	NR	NR	NR	Died	*Ureaplasma urealyticum* infection
Patient 3	M	—	12	549	NR	NR	NR	Died	*Ureaplasma urealyticum* infection
Patient 4	M	—	9	NR	NR	NR	NR	Survived	*Ureaplasma parvum* infection
Patient 5	F	—	10	>200	L, R	AR, LC, SB	IHD	Survived	*Ureaplasma parvum* infection
(2)		6	—						
Patient 1	M	—	—	1,597	L	NR	IHD	Died	Idiopathic
Patient 2	F	—	—	479	L, N	NR	CVVHD	Died	Idiopathic
Patient 3	M	—	—	366	L, N	AR, LC	IHD	Died	Idiopathic
Patient 4	M	—	—	289	L, ME, R	AR, LV, SP	IHD	Died	Idiopathic
Patient 5	F	—	—	304	L, ME, R	AR, LV, SP	IHD	Survived	Idiopathic
Patient 6	M	—	11	374	NR	NR	CVVHD	Died	Idiopathic
(12)	F	1	5	80	NR	NR	NR	Survived	*Ureaplasma*
(36)	M	1	29	246	L, ME, R	SP	NR	Survived	*Ureaplasma*
(37)	F	1	8	399	L, R	SB	CVVHD/HD	Died	Idiopathic
(18)	—	6	—						
Patient 1	M	—	18	312	L, ME, R	AR, LC, SB	CVVHD	Survived	Idiopathic
Patient 2	M	—	8	341	L, ME, R	AR, LC, SB, SP	CVVHD + IHD	Survived	Idiopathic
Patient 3	M	—	11	55	L, ME, R	AR, LC, SB, SP	CVVHD	Died	Idiopathic
Patient 4	M	—	45	189	L, ME, R	AR, LC, SB, SP	CVVHD	Died	Idiopathic
Patient 5	M	—	6	198	L, ME, R	AR, LC, SB	IHD	Survived	Idiopathic
(38)	M	1	8	830	L, R	NR	CRRT + HD	Died	*CPS I inhibition by VPA*
(39)	M	1	32	506	L, R	SB, SP	CVVHD	Survived	*NR*
(40)	—	2	—						
Patient 1	F	—	3	144	L, R, ME	LC, SB	MARS	Survived	Idiopathic
Patient 2	F	—	9	95	L, R, ME	NR	MARS/ECMO/PP/RRT	Survived	Idiopathic

AA, acetohydroxamic Acid; AR, arginine; CA, carbaglumic acid; CAVHD, continuous arteriovenous hemodialysis; CVVHD, continuous veno-venous hemodialysis; CPS I, carbamoyl phosphate synthase I; ECMO, extracorporeal membrane oxygenation; F, female; IHD, intermittent hemodialysis; L, lactulose; LC, levocarnitine; M, male; MARS, molecular adsorbent recirculating system; ME, metronidozole; N, neomycin; NR, not reported; PP, plasmapheresis; POD, Post-operative day; R, rifaximin; RRT, renal replacement therapy; SB, sodium benzoate; SP, sodium phenylacetate; VPA, valproic acid.

### Treatment and Outcome

The majority of patients who received bowel decontamination were administered lactulose, rifaximin, metronidazole, or neomycin. Besides arginine or levocarnitine, ammonia scavengers were used in at least 15 of the reported cases, with few cases using up to four different agents. Continuous veno-venous hemodialysis (CVVHD) was the primary RRT in 15 patients. Intermittent hemodialysis (iHD) was used in eight cases. In six patients, the combination of the two was used. One case used continuous arterio-venous hemodialysis (CAVHD), and two cases used a molecular adsorbent recirculating system (MARS) in combination with plasmapheresis, extracorporeal oxygenation (ECMO), and RRT.

The majority of reported etiology was idiopathic (26 cases), followed by Mycoplasma/ureaplasma infection (nine cases) and GS deficiency (two cases). One case attributed etiology to inhibition of carbamoyl phosphate synthase by valproic acid, and two others did not have etiology reported.

## Experience at Our Center

### Case 1

A 68-year-old man with end-stage idiopathic pulmonary fibrosis underwent bilateral sequential LT. The patient developed severe encephalopathy on post-op day 2. His clinical condition further deteriorated, and he went into distributive shock, requiring several vasopressors. Antimicrobial coverage was broadened to include levofloxacin, metronidazole, and micafungin. Baseline lab (Table 2) and computed tomography of the head, chest, and abdomen were unrevealing. Electroencephalogram was negative for seizures. Serum ammonia (NH_3_) level on post-op day six was 245 μmol/L, which was elevated from the immediate post-transplant level of 55 μmol/L (normal <60 μmol/L). A comprehensive workup for an infection that included blood cultures for urea-splitting organisms, bacteria, and fungi was negative. Serum ammonia continued to rise despite the implementation of RRT and aggressive bowel decontamination with lactulose and metronidazole. The patient’s distributive shock persisted. At this juncture, the possibility of a urea cycle (UC) disorder was considered, and intravenous sodium benzoate, sodium phenylacetate, and arginine were initiated. With the above therapies, the patient’s serum ammonia returned to normal over the next 3 days. This coincided with the normalization of his hemodynamics, serum lactate level, and resolution of his altered mental status. Unfortunately, the patient subsequently developed severe septic shock from a perforated cecum that required exploratory laparotomy, colectomy, and placement of ileostomy. Following this, a transjugular liver biopsy was performed for elevated liver enzymes, and this was complicated by hemorrhagic shock. At this point, the patient’s family withdrew care.

**TABLE 2:** Index patients’ laboratory results at presentation.

	Reference Range	Case 1	Case 2	Case 3	Case 4
Sodium	136–145 mmol/L	141	143	137	140
Potassium	3.3–5.1 mmol/L	4.6	4.2	4.8	3.9
Chloride	98–107 mmol/L	96 (L)	102	95	102
Carbon dioxide	22–30 mmol/L	29	24	31	28
Urea nitrogen	6–20 mg/dl	13	19	13	8
Creatinine	0.80–1.20 mg/dl	1.05	1	0.83	0.79
Glucose	65–99 mg/dl	125 (H)	92	319	87
Calcium	8.4–10.2 mg/dl	9.7	9.9	10	9.6
Anion gap	8–16 mmol/L	16	17	11	10
Total protein	6.4–8.3 g/dl	7.3	8	8	7.8
Albumin	3.5–5.0 g/dl	4.2	4.3	4.4	3.8
Phosphorus	2.7–4.5 mg/dl	3.0	5.2	2.3	3.5
AST	0–37 U/L	13	21	20	16
ALT	0–41 U/L	15	17	14	6
Total bilirubin	0.0–1.0 mg/dl	0.3	0.4	0.3	0.4
Alkaline phosphatase	35–129 U/L	78	45	104	88
Magnesium	1.5–2.8 mg/dl	1.5	1.8	2.1	1.9
WBC	4.0–10.0 thou/cu mm	12.3 (H)	8.3	19.5	9.6
Hemoglobin	13.0–16.5 g/dl	15.3	17.2	12.6	11.3
Platelet count	150–450 thou/cu mm	227	180	482	199

AST, aspartate aminotransferase; ALT, alanine aminotransferase; WBC, white blood cell.

### Case 2

A 64-year–old man with end-stage idiopathic pulmonary fibrosis, diabetes, and sleep apnea, underwent uncomplicated bilateral sequential LT. Cultures from the donor bronchus grew methicillin-sensitive staph aureus, which was treated with cefazolin for 7 days. Postoperative ammonia level on day 2 was 155 μmol/L with a follow-up level of 77 μmol/L. Levofloxacin to cover urea spitting organisms and metronidazole to provide bowel decontamination was started. Ammonia level decreased to 35 μmol/L on post-op day 3, and he was extubated the following day. However, the ammonia level increased to 77 μmol/L. Azithromycin was added for dual coverage of ureaplasma and mycoplasma. All sources of oral protein intake were stopped for 24 h. Enteral sodium phenylbutyrate, rifaximin, lactulose, and intravenous arginine in dextrose with micronutrients (Supplementary Material S2), and intravenous lipid as a source of calories were initiated. Serum amino acid profile and urine orotic acid levels that were checked to detect underlying UC disorders were negative (Table 3). The patient’s ammonia level continued to fluctuate between 60 and 80 μmol/L despite the above interventions. The patient was, however, asymptomatic without any signs of encephalopathy. Protein in the diet was introduced 48 h later (0.25 g/kg/d initially) and gradually increased to prevent catabolism. Ammonia level eventually started to downtrend with this multimodal therapy. The patient continued to improve and was ultimately discharged to a rehabilitation facility on post-transplant day 17.

**TABLE 3:** Metabolic profile and assay results.

	References range	Case 1	Case 2	Case 3	Case 4
Citrulline	10–60 mmol/L	27	16	13	32
Argininosuccinate	0–2 mmol/L	0	<2	<2	<2
Arginine	40–160 mmol/L	72	48	61	48
Ornithine	20–135 mmol/L	74	42	67	94
Aspartate	0–25 mmol/L	4	4	10	6
Glutamine	410–700 mmol/L	287	539	614	488
Urinary Orotic acid	0.2–1.5 mol/mol	Not tested	0.9	0.3	0.4
BAL or Blood PCR	NA	Neg	Neg	Neg	Neg
Mycoplasma Culture	NA	No sent	Neg	Neg	Neg
Ureaplasma Culture	NA	Not sent	Neg	neg	Neg
Specimen Type	NA	Not sent	Blood^a^	BAL Fluid^b^, (41,42)	Blood^a^

BAL, bronchoalveolar lavage, D, donor, NA, not applicable, Neg, Negative, PCR, polymerase chain reaction, R, recipient.

a Test performed in ARUP laboratories.

b Test performed in Mayo Clinic laboratories.

### Case 3

A 54-year-old woman with end-stage COPD received bilateral LT. The patient was successfully extubated on post-op day 1. However, she developed primary graft dysfunction grade 3 on post-op day two, requiring initiation of ECMO. During that time, she became confused and encephalopathic, and her ammonia level was found to be 122 μmol/L. She was started on levofloxacin, rifaximin, and doxycycline for empiric treatment of mycoplasma and ureaplasma infections. The PCR assays for these organisms, however came back negative. All protein in the diet was discontinued. Oral sodium phenylbutyrate and intravenous arginine, dextrorse, and carnitine were started (Supplementary Material S2). Ammonia levels continued to fluctuate between 100–140 μmol/L with a peak of 146 μmol/L on day 18 despite the above treatment regimen. Intermittent hemodialysis was started for 4 h daily with no appreciable change in the ammonia level. Oral sodium benzoate was added to the treatment. The duration of HD was increased to 6 h daily. CVVH was added in between the HD. These measures decreased the ammonia levels to an average of 40–60 μmol/L. The patient recovered and was discharged home.

### Case 4

A 64-year-old woman with rheumatoid arthritis-related interstitial lung disease underwent bilateral LT. The patient was extubated on day 1. She became febrile with elevated white blood cell count on post-op day 3. Ammonia level in the blood sample was elevated at 162 μmol/L. The patient was reintubated for respiratory failure on post-op day 4. Pressors were started, and antibiotics were broadened to include rifaximin, lactulose, azithromycin, levofloxacin, micafungin, metronidazole inhaled tobramycin. Cultures from both initial and follow-up bronchoalveolar lavages remained negative. Hyperammonemia protocol was initiated (Supplementary Material S2). All protein sources in the diet were stopped. Intravenous arginine in dextrose and oral sodium phenylbutyrate were started. Daily extended iHD for 6 h was launched with CVVHD in-between. The ammonia level decreased to 82 μmol/L. Ureaplasma and Mycoplasma PCR assays were negative (Table 3). The patient was discharged to the rehabilitation center on day 66 post-transplantation.

## Discussion

Whether previous cases reporting etiology as idiopathic or more recently ones attributed it to mollicutes, none took a metabolic focus to describe if HA impacted UC or GS pathways or reported aminoassay to better assess if UC or GS were implicated in HALT. Table 3; Figure 2 and Supplementary Material S3 describe our metabolic analyses and illustration of index cases.

Induction immunosuppression was the same in all four cases and included basiliximab administered on the day of surgery and a second dose on post-op day 4 per our center’s protocol (Table 4). As ammonia levels started to increase in the above cases, all sources of protein intake in the diet were stopped. Broad antimicrobial coverage targeting mycoplasma and ureaplasma was initiated. Despite that, ammonia continued to increase (Supplementary Material S3). Intravenous lipid and dextrose were used as a source of calories for the first 24–48 h post-diagnosis. This was followed by a gradual introduction of protein (starting at 0.25 g/kg and increased to target protein intake). The intravenous ammonia scavenger used in the first case was successfully replaced with oral agents in the other three cases. In patients with a rapid increase in ammonia levels, RRT was promptly instituted. In hemodynamically stable patients, iHD was preferred over CRRT. In patients who continued to have higher ammonia levels despite iHD, strategies that include a longer duration of iHD up to 6 hours and adding CRRT between iHD sessions were adopted. Amino acid profile (all four cases) and urinary Orotic acid (case 2, 3, and 4) were within normal limits or negative, indicating that urea cycle disorders (UCD) or metabolic diseases are less likely to be the underlying triggers (Table 2). Ureaplasma PCR in all the cases was negative. The increase in ammonia levels started on post-transplant days 11, 1, 3, and 4 (in cases 1, 2, 3, and 4, respectively). Ammonia level peaked at 245, 155, 146, and 176 μmol/L (cases 1, 2, 3, and 4, respectively). The use of oral or enteral ammonia scavenger instead of intravenous was effective and was associated with significant cost savings.

**TABLE 4:** Summary of patient characteristics, and clinical course.

	Case 1	Case 2	Case 3	Case 4
Age	68	64	54	64
Gender	M	M	F	F
Race	CA	CA	CA	AA
Presenting Symptoms	Encephalopathy, fever, shock/PRESS	Asymptomatic	Confusion	Unable to assess as patient intubated for respiratory failure
Transplant indication	IPF	IPF	PE/PH	RA ILD/IPF
Transplant type	BL	BL	BL	BL
CMV Donor D/Recipient R	−/−	+/−	−/+	+/−
EBV	I/+	+/+	+/+	+/+
Induction	B	B	B	B
Maintenance	MMF, MP, T	MMF, MP, T	MMF, MP, T	MMF, MP, T
Post Op complications	AMS/Sepsis/bleeding/MV/MOF	A fib, Left subclavian DVT	Primary graft dysfunction grade 3	Trached but weaned, clam shell wound dehiscence
		Wound dehiscence s/p debridement and wound vac placement		
—	—	—	ECMO	ECMO
Initial ammonia value^a^	55	29	54	39
Day ammonia Peaked	10	6	18	10
Peak ammonia value	245	155	146	176
Antimicrobial agents at time of diagnosis	AZ, DO	AZ, L	AZ, DO, L	AZ, L
	CE/V/ME/L/MI	ME	CE/V/	CE/V/ME/ME

AA, african american; A Fib, Atrial fibrillation; AMS, altered mental status; AZ, azithromycin; B, basiliximab; BL, bilateral; CA, caucasian american; CE, cefepime; CMV, cytomegalovirus, D, donor, DO, doxycycline; DVT, deep vein thrombosis; EBV, epstein barr virus; ECMO, extracorporeal membrane oxygenation; F, female; H, hispanic; I, intermediate; IPF, idiopathic pulmonary fibrosis; L, levofloxacin; M, male; ME, metronidazole; MI, micafungin; MMF, mycophenolate mofetil; MP, methylprednisolone; MV, mechanical ventilation; MOF, multiorgan failure; PE, pulmonary emphysema; PH, pulmonary hypertension; PRESS, posterior reversible encephalopathy syndrome; RA ILD, rheumatoid arthritis interstitial lung disease; R, recipient; T, tacrolimus; V, vancomycin.

a Ammonia level at the day of transplantation.

The incidence of HALT in our institution (3.3%) is consistent with previous reports (1–3). Our mean peak ammonia level was 185 μmol/L, much lower than reported previously. The median days to peak ammonia level was also shorter (11 days compared to 14.1). These differences could be related to early recognition and prompt institution of a multimodal treatment plan (Supplementary Material S2).

## Etiology

### Underlying Urea Cycle Disorder

HALT was initially thought to be caused by unmasking of partial UC defect under the metabolic stress of transplantation (1,13,14). However, in all our four cases, amino acid profile and orotic acid results did not suggest the presence of a UCD. Glutamine, citrulline, arginine, ornithine in all cases, and orotic acid levels in cases 2, 3, and 4 were normal. Moreover, there were no reported cases of UCDs in patients with HALT in the lung transplant literature. In particular, quantitative analysis of UC enzyme expression in HALT’s fatal case showed no evidence of loss of urea cycle enzyme expression (1). Moreover, our cases’ glutamine levels were either normal or below normal, indicating the less likelihood of UCDs as an etiology in this patient population (19).

### Ureaplasma/Mycoplasma

Bharat et al. utilized specialized culture, polymerase chain reaction, and molecular resistance profiling to provide evidence supporting a causal relationship between *Ureaplasma* infections and HA in LT recipients (11,12). Empiric dual antibiotic coverage that includes levofloxacin, azithromycin, and or doxycycline was initiated in all four cases. Despite the timely initiation of antibiotics, ammonia levels continued to rise. Ureaplasma and mycoplasma PCR assays from BAL or blood were sent in all but the first case (Table 2) and were negative. These tests are performed in few specialized labs in the country. Moreover, the turnaround time for the results ranges from 3 days to a week. Hence the clinician will have to start empiric antibiotics even before the results of the tests are available.

### Medication-Induced

Seizures resulting from intravenous calcineurin use and leading to the development of HA have been described. None of our cases developed seizures while on calcineurin inhibitors.

### Hepatic Glutamine Synthetase Deficiency

Glutamine synthetase (GS) is a cytosolic enzyme that catalyzes ammonia and glutamate condensation to produce glutamine, which is a substrate for various metabolic pathways and is essential for many organs. GS also plays a crucial role in 1) protecting the neurons by capturing ammonia and glutamate in the glial cells; and 2) supplying glutamine for glutamate and GABA synthesis in the glutamine-glutamate-GABA cycle, regulating the excitatory and inhibitory synaptic transmission of neurons (20–22).

Urea cycle-ammonia detoxifying effect accounts for only 35%. Using GS knockout/liver, control mice, and stepwise increments of enterally infused ammonia, Hakvoort et al. showed that the other 35% of ammonia is detoxified by GS while the remaining 30% is not cleared by the liver (23). They further showed, through genetic and pharmacologic approaches to modulate GS activity, that stepwise increments detoxification of intravenously infused ammonia is almost totally dependent on GS activity (23). GS deficiency causes only mild to moderate hyperammonemia (24,25), as it might be the case in some of the HALT. Previous studies have shown decreases in hepatic GS enzyme activity to 12% and 28% of the mean value of controls in two cases (1,19). In one patient in which we were able to obtain liver tissue, there was near-complete loss of hepatic GS expression (Figure 2). Further supporting a critical role of hepatic GS, rather than a UCD in the pathogenesis of HALT. Analysis of systemic amino acid profiles in several patients in our case series showed normal amino acid profiles, which is atypical for a primary urea cycle disorder. Thus, to our knowledge, every case of HALT, which has examined hepatic GS expression, has identified substantial hepatic GS deficiency. Therefore, these findings are consistent with the possibility that LT induces a transient down-regulation of hepatic GS expression in susceptible individuals, leading to the development of HA.

## Management

### Enteral Versus Intravenous Therapy

To our knowledge, this is the first case series reporting the successful use of combination oral ammonia scavengers in patients with HALT. In our second, third, and fourth cases, we successfully used oral sodium phenylbutyrate (Supplementary Material S2) instead of intravenous sodium benzoate/sodium phenylacetate. In the third case, we added oral benzoate at a dose of 5.5 g/m^2^ to sodium phenylbutyrate for more aggressive clearing of ammonia in addition to dialysis and observed no identifiable complication. This has not been described before, as it was thought that both medications have similar mechanisms of action. Diarrhea might cause suboptimal absorption of the ammonia scavengers. Diarrhea is common in HALT patients due to the use of bowel decontamination agents. Similarly, CRRT and extended iHD might result in augmented clearance of the medication. Due to the above two concerns, sodium benzoate was added. We have also added carnitine and micronutrients to the arginine-dextrose to help substrate utilization (see below).

### Role of Dialysis

Dialysis and dialytic modalities are an integral part of HALT management, though in some cases, dialysis may not be required. Currently, there is no consensus of appropriate timing to initiate dialysis, but some clinicians suggest considering dialysis if the ammonia level exceeds three times the upper limit of normal in the absence of liver disease (26). The main goal is to reduce the ammonia level as quickly as possible. Lag time between diagnosis and initiation of dialysis may contribute to adverse outcomes (27). Intermittent hemodialysis with a large surface area dialyzer is a more efficient modality over CRRT and peritoneal dialysis or charcoal hemoperfusion. Extended dialysis session of ≥6 h, a blood flow rate of 400 ml/min, and a fluid flow rate of 800 ml/min is more efficient in clearing ammonia. If iHD is not an option due to hemodynamic instability, sustained low-efficiency dialysis or continuous veno-venous hemodialysis at the rate of 250 ml/kg/h and 40–50 ml/kg/h, respectively, should be considered with the highest possible blood flows (27). High flux daily HD, in addition to other adjunct therapies, should be considered.

### Early Diagnosis

At our center, we routinely check daily ammonia levels for the first-week post LT and also when there is any sign of mental status change, thus diagnosing this condition at a very early stage. Early diagnosis strategy has enabled us to institute early treatment, thus preventing ammonia levels from getting very high. This has resulted in better survival of our patients.

### Role of Micronutrients

The direct and toxic effects of HA on the astrocytes within the brain (such as oxidative/nitrosative stress due to disturbance of the NO pathway), creatine deficiency, and inhibition of the tricarboxylic acid cycle have been described. These toxic effects can lead to secondary mitochondrial failure, and thus, energy deficiency; hence adding micronutrients to arginine and intravenous continuous dextrose infusion may help better substrate utilization (Supplementary Material S2) (21,28,29).

## Perspective

While some reports have described the casual association between mollicutes and HA (11,12,14–17,30), there is no conclusive evidence that this is the only possible etiology for HALT. We initiated dual antibiotics targeting these mollicutes very early in our case series. Despite the continued use of these antimicrobials, we witnessed a worsening in ammonia levels prompting multimodal interventions. Additionally, the tests for all the mollicutes have been negative (Table 3, Supplementary Material S3). All these seem to indicate that there is possibly more than one etiology for HALT. The metabolic and biochemical analyses of our index cases are unique to our study to show LT effect on UC as described above (Table 3). while Baharat et al. (11), showed disseminated ureaplasma/mycoplasma as the cause for HALT, it is unclear how these infections would impact UC or GS pathways.

While mortality rate of patients who develop HA is historically very high. Our case “series” mortality was lower, indicating early detection and early multimodal treatment might have contributed to improved survival. However, such an approach may also result in slightly more patients being treated with this multimodal strategy. Since historically, the mortality with this disease has been so high, the benefits of treatments outweigh the decision to delay treatment or the decision not to treat. Our center has developed a very safe and comprehensive protocol to treat this disease with highly favorable results. As there is no single exact etiology for this condition and probably several mechanisms at play, a multimodal treatment approach appears to be the best.

The cause of HALT remains elusive. It may be related to reduced GS activity and unmasking of partial UC defects. Early diagnosis of this syndrome and implementing a multidimensional therapeutic approach is paramount for a successful outcome. A very efficacious and cost-effective successful multimodal strategy for the treatment of HALT is described here. Practical issues include provider and nursing education and proper handling of the ammonia specimen.

Based on our experience, we suggest early testing and close monitoring of ammonia levels. Multimodal strategies to manage HALT include stopping all protein in the diet for the first 24 h, early initiation of ammonia scavenger medications, dialysis, and broad-spectrum antibiotic with mycoplasma and ureaplasma coverage and utilization of dextrose, lipid, and micronutrients as a source of calories in the acute phase. We believe that this will improve the survival of patients with this condition.
