# Corrigendum: Hyperammonemia After Lung Transplantation: Systematic Review and a Mini Case Series

**DOI:** 10.3389/ti.2022.10670

**Published:** 2022-07-08

**Authors:** Amir Y. Kamel, Amir M. Emitazjoo, Lauren Adkins, Abbas Shahmohammadi, Hassan Alnuaimat, Andres Pelaez, Tiago Machuca, Mauricio Pipkin, Hyun-Wook Lee, I. David Weiner, Satish Chandrashekaran

**Affiliations:** ^1^ Department of Pharmacy, UF Health Shands Hospital, College of Pharmacy, University of Florida, Gainesville, FL, United States; ^2^ Division of Pulmonary, Critical Care and Sleep Medicine, UF Lung Transplant Program, College of Medicine, University of Florida Health Hospital, Gainesville, FL, United States; ^3^ College of Pharmacy Liaison Librarian, Health Science Center Libraries, Gainesville, FL, United States; ^4^ Division of Cardiothoracic Surgery, UF Lung Transplant Program, University of Florida Health Hospital, College of Medicine, University of Florida, Gainesville, FL, United States; ^5^ Division of Nephrology, Hypertension and Renal Transplantation, College of Medicine, University of Florida, Gainesville, FL, United States; ^6^ North Florida/South Georgia Veterans Health System, Gainesville, FL, United States

**Keywords:** hyperammonemia, lung transplantation, ammonia, glutamine synthetase, urea cycle, ammonia scavengers, mollicutes

In the original article, we neglected to include the funder NIDDK, R01-DK107798, for author IW. The updated funding statement is “These studies were supported by funds from NIDDK R01-DK-107798.”

An author name was incorrectly spelled as “David I. Weiner.” The correct spelling is “I. David Weiner.”

The authors apologize for these errors and state that this does not change the scientific conclusions of the article in any way. The original article has been updated.

