# Supplementary figures and images for: Hyperammonemia After Lung Transplantation: Systematic Review and a Mini Case Series
Source: Transpl Int. 2022 May 3;35:10433. doi: 10.3389/ti.2022.10433 (PMC9128545; doi:10.3389/ti.2022.10433)

## Supplemental 3 Cases and Analyses Review

### Case 1

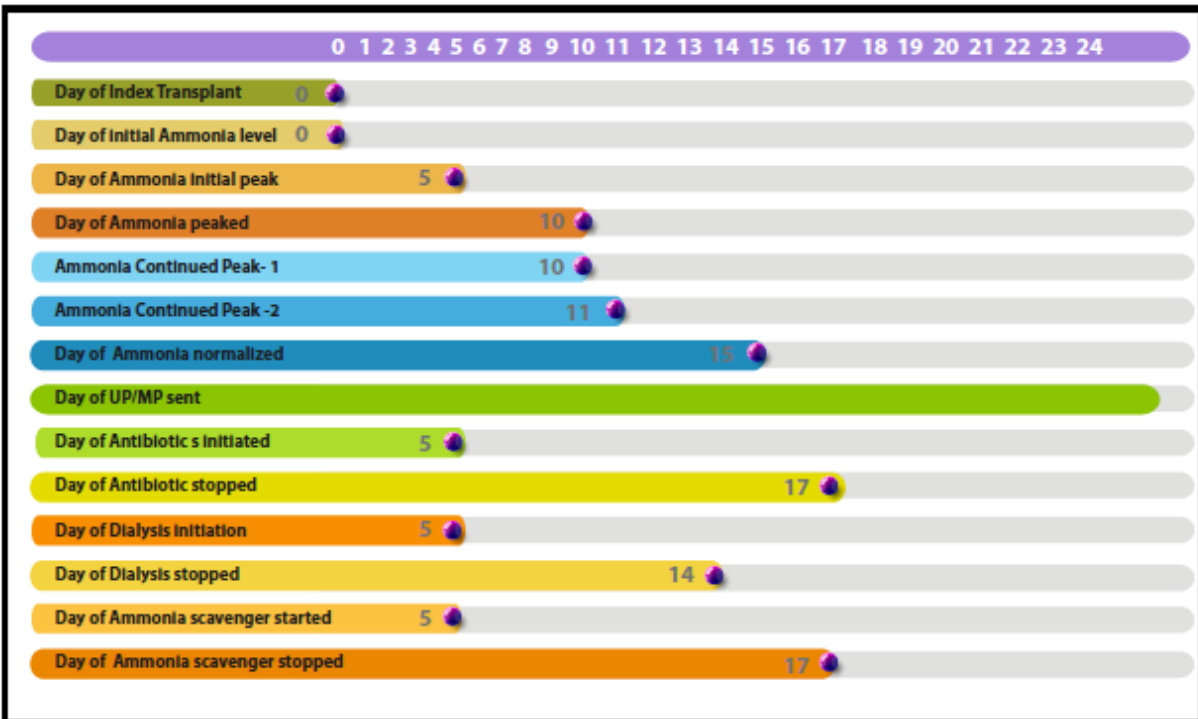

### Case 2

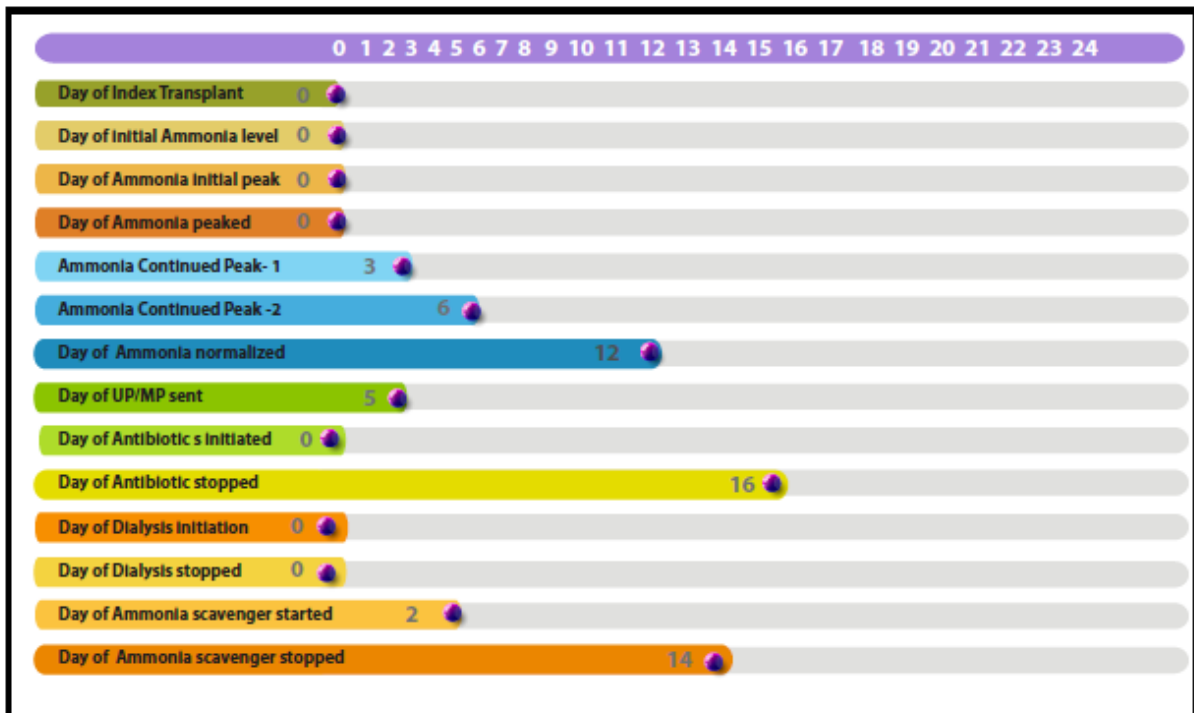

### Case 3

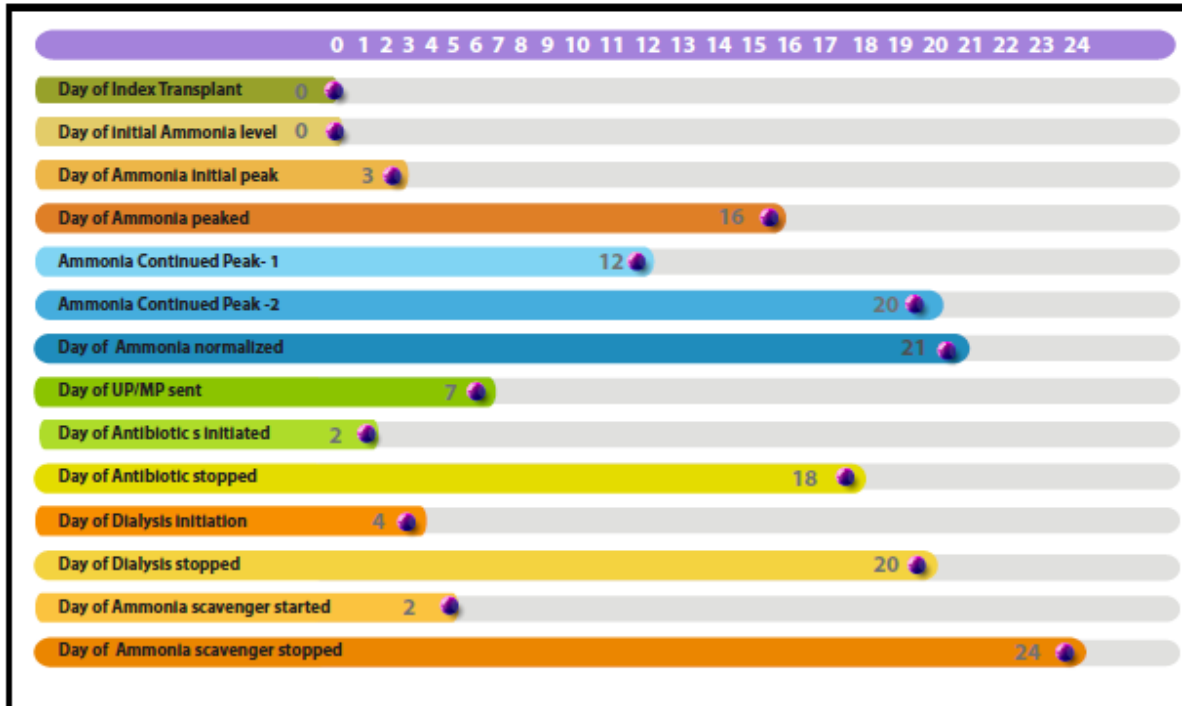

### Case 4

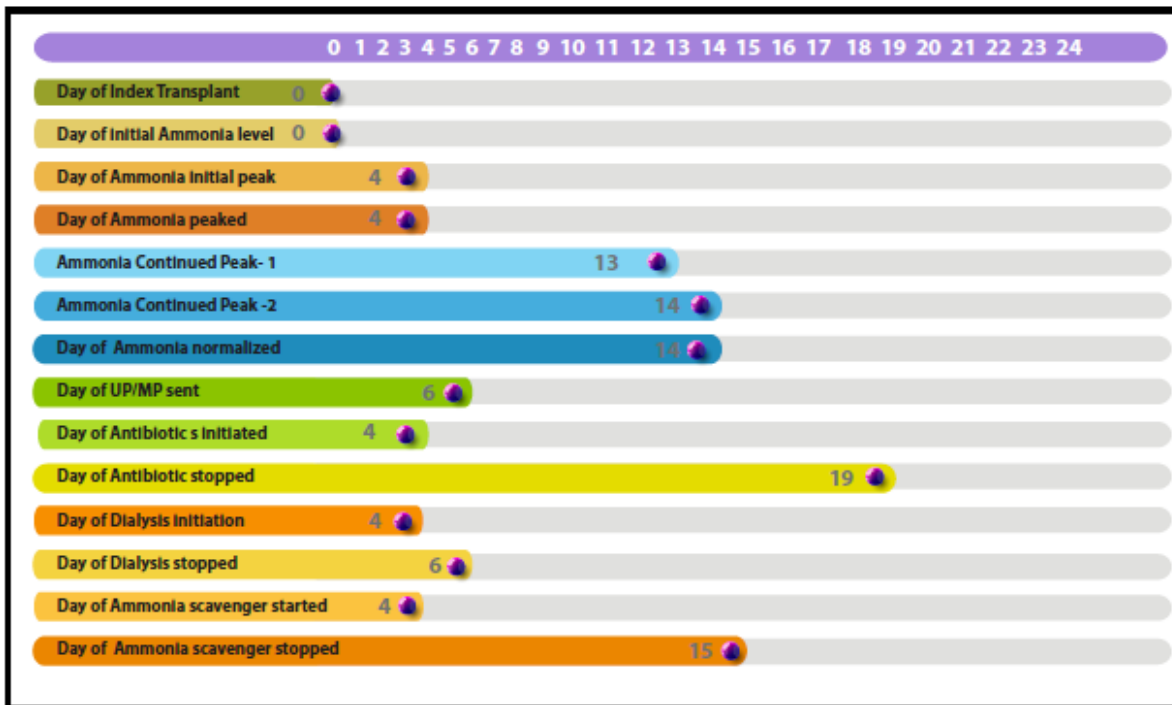

Supplement: Supplementary file 1 [file DataSheet3.pdf]
