# Supplementary material for: Hyperammonemia After Lung Transplantation: Systematic Review and a Mini Case Series
Source: Transpl Int. 2022 May 3;35:10433. doi: 10.3389/ti.2022.10433 (PMC9128545; doi:10.3389/ti.2022.10433)
Supplement: Supplementary file 2 [file DataSheet1.PDF]

Supplement 1: Search Strategy for Hyperammonemia after Lung Transplantation.

Search conducted on July 8th, 2019 and an updating search conducted on April 27th, 2020.

No.	Search Query	Filters
1	("Hyperammonemia"[Mesh] OR "Ammonia"[Mesh] OR hyperammonemi*[tiab] OR Hyperammonaemi*[tiab] OR ammonia*[tiab])	
2	("Lung Transplantation"[Mesh] OR "lung transplantation"[tiab] OR "lung transplantations"[tiab] OR "Lung Grafting"[tiab] OR "Heart-Lung Transplantation"[Mesh] OR "Heart-Lung Transplantation"[tiab] OR "Heart Lung Transplantation"[tiab] OR "Heart-Lung Transplantations"[tiab] OR "Heart-Lung grafting"[tiab] OR ((lung*) AND transplant*))	
3	#1 AND #2	Publication Year: 1/01/1995 to 7/8/2019<br><br>Language: English

Original Number of Citations

Database	Search 1	Search 2	Search 3
PubMed	85	9	4
Embase	113	2	39
Web of Science	120	3	1
Cochrane Library	12	1	1
Clinical Trials.gov	142	3	6
CINAHL	11	1	2
International Pharmaceutical Abstracts (IPA)	1	9	0
Total Number of Results	484	28	53
Duplicates Removed	232	12	5

Total Number of Duplicates Removed: 249

Database	Search 1	Search 2	Search 3
PubMed	50	4	3
Embase	96	2	39
Web of Science	36		0
Cochrane Library	11	1	1
Clinical Trials.gov	55		5
CINAHL	4		0

International Pharmaceutical Abstracts (IPA)	0	9	0
Total Number of Results	252	16	48

Combined number of results: 316
