# Supplementary material for: Hyperammonemia After Lung Transplantation: Systematic Review and a Mini Case Series
Source: Transpl Int. 2022 May 3;35:10433. doi: 10.3389/ti.2022.10433 (PMC9128545; doi:10.3389/ti.2022.10433)
Supplement: Supplementary file 3 [file DataSheet2.docx]

# Supplement 2: Hyperammonemia after Lung Transplant [2,11,18,27,31,42]

**Serum ammonia and other lab monitoring**

- Ammonia level to be sent immediately after transplant
  - Tourniquet should not be used when drawing sample, this may falsely elevate level.
  - Sample should be stored on ice and delivered in person to the lab immediately after draw (Don’t send using tubing system).
  - Sample should be processed as soon as possible, within 30 minutes
- If elevated (> 60 mcmol/L)
  - Ammonia should be repeated every 6 hours from an arterial sample preferably
  - When 2 consecutive levels stable and < 60 mcmol/L, decrease frequency to every 8 hours
- Other labs in setting of hyperammonemia
  - Plasma aminoacid (AA), urinary AA, urinary organic acid, urinary orotic acid, acylcarnitine profile, organic acid profiles x1
  - Ureaplasma and Mycoplasma culture or PCR x1
  - LFTs, INR, BMP, CBC with differential daily
  - Closely monitor acid/base status while on therapy. An anion gap of ≥15 mcmol/L or increase of ≥ 6 mcmol/L suggests nitrogen scavenger accumulation

**Hyperammonemia treatment**

Serum ammonia > 60 mcmol/L:

- Discontinue all sources of protein intake (including tube feeds) for first 24-48 hours
  - After 48 hours, resume protein at 0.25 g/kg, then gradually increase to goal per RD recommendations
- Consider renal replacement therapy
  - Consult nephrology
  - iHD preferred – large surface area dialyzers, up to Qb 400 mL/min and Qd 800 mL/min with extended dialysis time of at least 6 hours
- Ensure patient has adequate bowel regimen as hyperammonia has been reported with constipation
- Initiate following medications:
  - Antibiotics to cover Ureaplasma/Mycoplasma (consider dual coverage):
    - Azithromycin 500 mg PO/IV q24h
    - Levofloxacin 750 mg PO/IV q24h
    - Doxycycline 100 mg PO/IV q12h
  - Nitrogen scavengers:
    - Sodium Phenylbutyrate (Buphenyl) 9-13 g/m^2^ PO divided q6h (not to exceed 20 g/day)
      - If ammonia not controlled after 72 hours , consider addition of Sodium benzoate 5.5 g/m^2^ PO divided q6h
    - Arginine HCl 10%: 200 mg/kg/day IV continuous as initial dose
      - After 48 hours, consider decreasing for maintenance dose (4 g/m^2^)
    - Levocarnitine 100 mg/kg/day IV continuous or divided every 4 hours
  - Dextrose 10% (+/- 0.45% NaCl or Na acetate depending on acid/base status) 25 mL/kg/day (2.5 g/kg/day) continuous infusion
    - Consider insulin infusion if needed to control blood glucose
  - Can combine dextrose, arginine and levocarnitine as continuous infusion (in sterile water) with the following additives (for 5 days):
    - Thiamine 300 mg/24 hours
    - Pyridoxine 50 mg/24 hours
    - Zinc 10 mg/24 hours
    - Selenium 150 mcg/24 hours
    - After initial 48 hours, may add 0.25 g/kg protein & advance as tolerated
  - Intralipid 20% 250 mL q12 hours continuous
  - Bowel decontamination:
    - Lactulose 30 g PO q8h
      - Consider decreasing frequency or holding to allow for PO absorption of nitrogen scavengers if excessive diarrhea (i.e. more than 3 bowel movements per day)
    - Rifaximin 550 mg PO q12h
    - Metronidazole 500 mg PO/IV q8h

Serum ammonia >500 mcmol/L OR two consecutive results >250 mcmol/L:

- Initiate renal replacement therapy (iHD preferred – large surface area dialyzers, up to Qb 400 mL/min and Qd 800 mL/min with extended dialysis time of at least 6 hours)
- Consult Genetics and Metabolism
- With Pediatric Genetics and Metabolism approval, initiate IV nitrogen scavenger (in place of Sodium Phenylbutyrate PO):
  - Sodium pheylacetate and sodium benzoate (Ammonul) 5.5 g/m^2^ IV over 90 min then 5.5 g/m^2^ IV continuous infusion daily
